# Roles of bile acids in enteric virus replication

**DOI:** 10.1186/s44149-021-00003-x

**Published:** 2021-04-23

**Authors:** Fanzhi Kong, Linda J. Saif, Qiuhong Wang

**Affiliations:** 1 Center for Food Animal Health, Department of Animal Sciences, College of Food, Agricultural and Environmental Sciences, The Ohio State University, Wooster, OH USA; 2 College of Animal Science and Veterinary Medicine, Heilongjiang Bayi Agricultural University, No. 5 Xinfeng Road, Sartu District, Daqing, China; 3 Department of Veterinary Preventive Medicine, College of Veterinary Medicine, The Ohio State University, Columbus, OH USA

**Keywords:** Bile acids, Coronavirus, Calicivirus, Rotavirus, Norovirus, Sapovirus

## Abstract

Bile acids (BAs) are evolutionally conserved molecules synthesized in the liver from cholesterol to facilitating the absorption of fat-soluble nutrients. In the intestines, where enteric viruses replicate, BAs also act as signaling molecules that modulate various biological functions *via* activation of specific receptors and cell signaling pathways. To date, BAs present either pro-viral or anti-viral effects for the replication of enteric viruses *in vivo* and *in vitro*. In this review, we summarized current information on biosynthesis, transportation and metabolism of BAs and the role of BAs in replication of enteric caliciviruses, rotaviruses, and coronaviruses. We also discussed the application of BAs for cell culture adaptation of fastidious enteric caliciviruses and control of virus infection, which may provide novel insights into the development of antivirals and/or disinfectants for enteric viruses.

## Introduction

Bile acids (BAs) are a large family of molecules that have a steroidal structure and are synthesized from cholesterol in the liver and actively secreted along with cholesterol, bilirubin, and phospholipids into bile. Serving as important signaling molecules, BAs have important physiological functions, including elimination of cholesterol, absorption of fat and fat-soluble vitamins, and regulation of gut microbiome (Molinaro et al. 2018; Monte et al. 2009; Tian et al. 2020; Wahlstrom et al. 2016). Plasma membrane-bound G protein-coupled receptors (GPCRs) and nuclear receptors are the two types of BA-activated receptors (Fiorucci et al. 2020). GPCRs include G protein-coupled BA receptor 1 (Kawamata et al. 2003), muscarinic receptor 2 (Cheng et al. 2002) and sphingosin-1-phosphate-2 (S1PR2) (Nagahashi et al. 2016). Nuclear receptors include farnesoid X receptor (FXR, also known as nuclear receptor subfamily 1, group H, member 4, NR1H4), pregnane X receptor, constitutive androstane receptor, vitamin D receptor, and small heterodimer partner (Shin and Wang 2019). In liver, BAs inhibit their own synthesis; in liver and intestine they regulate lipid and glucose homeostasis and suppress inflammation and fibrogenesis (Chiang 2009; Gonzalez-Regueiro et al. 2017; Li et al. 2017a; Namisaki et al. 2016; Pathil et al. 2014; Sinal et al. 2000). A disorder of BA metabolism results in severe pathological outcomes, such as cholestasis, hepatic steatosis, hepatic fibrosis, and liver tumors (Arab et al. 2017a; Arab et al. 2017b; Suga et al. 2019; Xie et al. 2016). Recently, research interest in the role of BAs in enteric virus replication has surged. In this review, we summarized biosynthesis, transport, and metabolism of BAs, discussed the roles of BAs in regulating enteric virus replication and the use of BAs for cell culture adaptation of fastidious enteric caliciviruses.

## Biosynthesis, transportation and metabolism of BAs

**Fig. 1:**
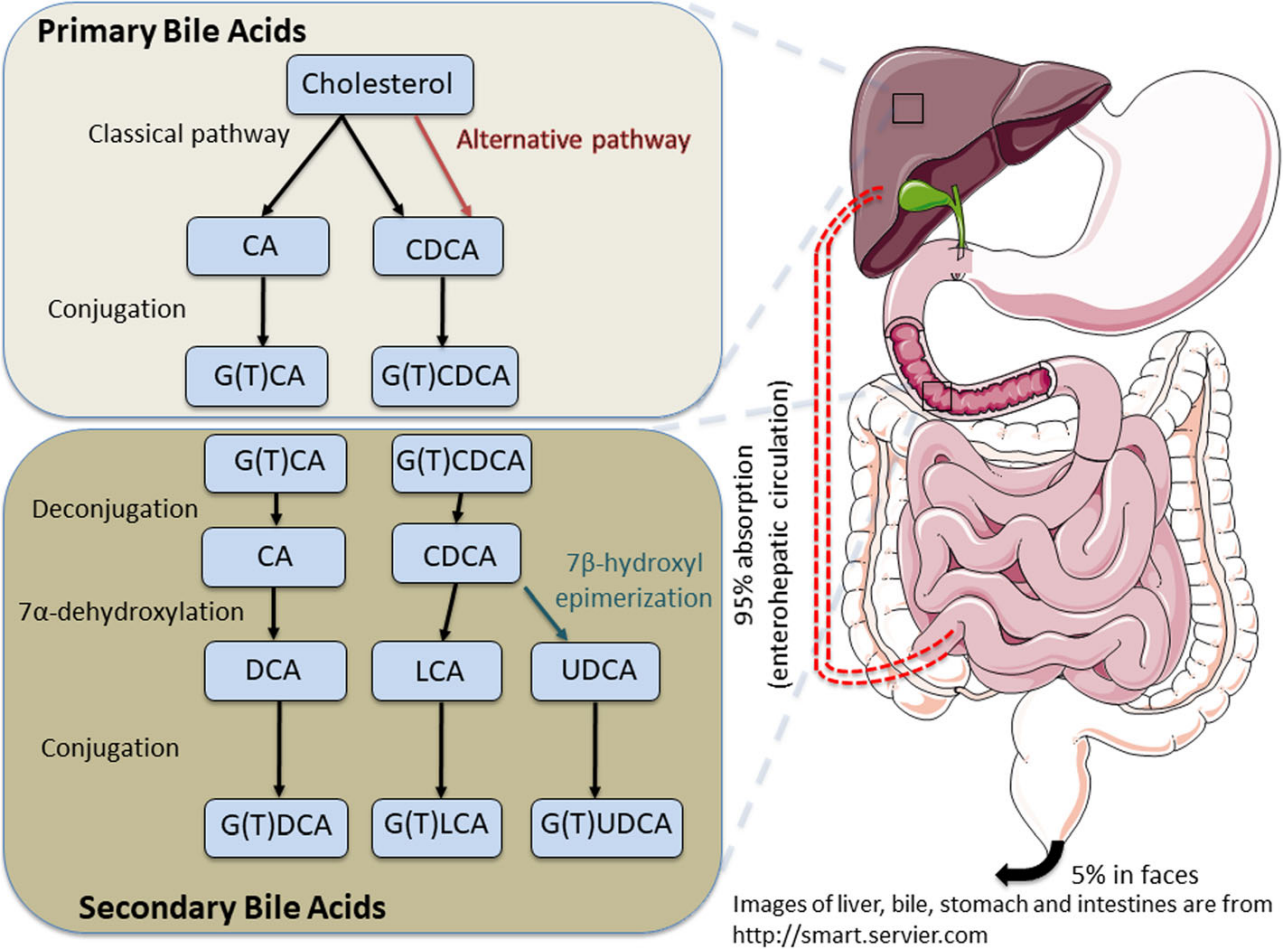
Bile acid (BA) biosynthesis and metabolism. Schematic representation of synthetic pathways of primary BAs in hepatocytes (tawny color) and secondary BAs in the intestine (dark brown color). The formation of BAs occurs in the liver via 2 pathways: the classical (or neutral) and the alternative (or acidic) pathways. BAs in the liver are then conjugated with glycine (G) or taurine (T). Primary BAs are metabolized by certain gut bacteria by deconjugation, dehydroxylation, conjugation, and epimerization, generating secondary BAs. The majority of BAs in the gut (90-95%) are reabsorbed in the ileum and recirculate to the liver through the portal vein. The remaining BAs are eliminated through the feces. CA, cholic acid. DCA, deoxycholic acid. CDCA, chenodeoxycholic acid. LCA, lithocholic acid. UDCA, ursodeoxycholic acid

Biosynthesis of BAs occurs in hepatocytes via cytochrome P450 (CYP)­ mediated oxidation of cholesterol through 2 biosynthetic pathways, the classical and alternative pathways (Fig. 1) (Chiang 1998). Primary BAs, cholic acid (CA) and chenodeoxycholic acid (CDCA) are synthesized from cholesterol in the liver through a series of enzyme cascades. Following the synthesis of CA and CDCA, some of them are conjugated with either taurine or glycine to form taurocholic acid (TCA), taurochenodeoxycholic acid (TCDCA), glycocholic acid (GCA) or glycochenodeoxycholic acid (GCDCA) in the liver (Fig. 1) (Russell 2003). Simultaneously, primary BAs are secreted into bile canaliculus from the liver. Bile containing BAs can be secreted directly into duodenum or stored and concentrated in the gallbladder. Acidic and fatty chyme causes the enteroendocrine I cells to secrete cholecystokinin into systemic circulation to stimulate the gallbladder to contract and secrete bile into the duodenum.

In the intestine, microbial enzymes from gut bacteria metabolize BAs. Primary unconjugated BAs, CA and CDCA are transformed by 7α-dehydroxylation into secondary unconjugated BAs, deoxycholic acid (DCA) and lithocholic acid (LCA), respectively (Fig. 1). The primary glycine or taurine conjugated CA (GCA or TCA) and CDCA (GCDCA or TCDCA) can be deconjugated by bile salt hydrolases to return to CA and CDCA, respectively. Epimerization of hydroxyl groups of CDCA by hydroxysteroid dehydrogenases of intestinal bacteria leads to the formation of ursodeoxycholic acid (UDCA). The secondary unconjugated BAs in the intestines are subsequently conjugated with glycine or taurine to form GDCA or TDCA, GLCA or TLCA, and GUDCA or TUDCA (Chiang 2013). At the distal ileum, approximately 95% intestinal BAs are reabsorbed by apical sodium-dependent BA transporter (ASBT, also known as SLC10A2) into enterocytes and secreted into the portal vein *via* basolateral BA transporters, including organic solute transporter subunit α (OSTα)/OSTβ complex. BAs are then taken up into hepatocytes by sodium/taurocholate co-transporting polypeptide (NTCP, also known as SLC10A1) and organic anion-transporting polypeptide 1 (Dawson et al. 2009; Trauner and Boyer 2003). There are alternative excretion routes for BAs to enter systemic circulation, such as those *via* multidrug resistance-associated protein 3 (MRP3) and MRP4 (Li et al. 2017b). The remaining 5% BAs are lost in feces or used by the intestinal microbiota (Ridlon et al. 2006). Certain gut bacteria metabolize BAs and transform them in the intestine into secondary unconjugated or conjugated forms with different biologic activities (Tian et al. 2020; Wahlstrom et al. 2016). These bacteria influence gut microbiome composition which is also shaped by diet and host factors. The impact of microbiome and BAs interactions on enteric viral infections is largely unexplored.

Biosynthesis of BAs is negatively regulated by FXR through a feedback mechanism to limit BAs accumulation in the liver (Goodwin et al. 2000; Lu et al. 2000). CA and CDCA, as well as DCA and LCA but to a smaller extent, are the endogenous ligands for nuclear receptor FXR (Makishima et al. 1999; Parks et al. 1999; Wang et al. 1999). Activation of ileal FXR, leading to down-regulation of ASBT and up-regulation of intestinal BA-binding proteins OSTα and OSTβ, promotes recycling of BAs to the liver (Chen et al. 2003; Landrier et al. 2006; Plass et al. 2002; Zollner et al. 2006). The circulation process, by which BAs are secreted from the liver into the intestine, reabsorbed in the ileum, and recycled to the liver *via* the portal vein, is termed enterohepatic circulation. In humans, a BA pool of about 3 g (90–95% of total BAs) is recycled between the gut and the liver approximately 8 times per day, with only 0.2–0.6 g of *de novo* synthesized BAs being produced per day to maintain a stable pool of BAs (Chiang 2009).

## The role of BAs in cell culture adaptation of enteric caliciviruses and in viral replication

Caliciviruses are small, round, non-enveloped viruses with a diameter of 27–35 nm. They possess a single-stranded, positive sense RNA genome (Prasad et al. 1999). Noroviruses and sapoviruses are members of caliciviruses and cause gastroenteritis in humans and animals (Green 2007; Oka et al. 2015). It had been difficult to study the pathogenesis and viral replication of human noroviruses (HuNoVs) and human sapoviruses (HuSaVs) due to the lack of a cell culture system until last decade. The fist cell culture-adapted enteric calicivirus was porcine enteric calicivirus (PEC) Cowden strain, a porcine genogroup III sapovirus. It was first isolated in primary porcine kidney cells (Flynn and Saif 1988) and subsequently in continuous porcine kidney cell line (LLC-PK1 cells) in the presence of an intestinal content (IC) preparation (Parwani et al. 1991). IC effects on the growth of PEC in cell culture were initially associated with the induction of a protein kinase A (PKA) signaling pathway (Chang et al. 2002). Subsequent studies revealed that bile and BAs functioned as active factors in IC and are essential for PEC growth in the continuous cell line LLC-PK1. In LLC-PK1 cells, BAs induced an increase in cyclic adenosine monophosphate (cAMP) concentration, which was associated with down-regulation of interferon (IFN)-mediated signal transducer and activator of transcription 1 (STAT1) activation, a key element in innate immunity (Chang et al. 2004). Further, Shivanna et al. found that GCDCA was critical for PEC escape from the endosomes into the cytoplasm by inducing acidification of endosomes and subsequent activation of the endosomal/lysosome-enzyme acid sphingomyelinase (ASM), and the production of ceramide to initiate viral replication (Shivanna et al. 2014, 2015). BA transporters, including ABST and NTCP, are also involved in exerting effects of BAs on PEC replication in cells. The pioneering discovery of requirement and mechanisms of certain BAs in the replication of PEC *in vitro* is very significant because it prompted scientists to culture HuNoVs and HuSaVs by using BAs.

HuNoVs are the leading cause of sporadic and epidemic gastroenteritis in all ages worldwide (Ahmed et al. 2014; Pires et al. 2015; Ramani et al. 2014). They are the major etiological agent of foodborne and waterborne gastroenteritis outbreaks. Recently, scientists found that HuNoVs target the intestinal epithelial of duodenum, jejunum and ileum (Green et al. 2020; Karandikar et al. 2016). More interestingly, Green et al. found that HuNoV replicated specifically in the enteroendocrine epithelial cells of biopsy samples collected from immunocompromised patients (Green et al. 2020). Also, significant progress has been made in propagation of HuNoVs *in vitro*. HuNoV replication in human B cells was facilitated by histo-blood group antigen (HBGA)-expressing enteric bacteria (Jones et al. 2014). Although cellular receptor for HuNoV entry is still unknown, HGBAs are considered as binding factors necessary to initiate infection. Later, scientists found that replication of certain HuNoV strains in human intestinal stem cell-derived enteroids (HIEs) and human induced pluripotent stem cells (iPSCs)-derived intestinal organoids was dependent on (or enhanced by) BAs (Ettayebi et al. 2016; Sato et al. 2019). Although HuNoV could infect three-dimensional cultured Caco-2 cells (Straub et al. 2011) and a clone of Caco-2 cells (C2BBe1) (Straub et al. 2013), reproducibility remains problematic (Takanashi et al. 2014). Interestingly, bile was involved in HuNoV replication in the enteroids and intestinal organoids in a strain-dependent manner and functioned at an early stage of infection, with its effects on the cells, but not the virus (Ettayebi et al. 2016; Sato et al. 2019). Recent studies have confirmed no binding of BAs (GCA, GCDCA, TCA or TCDCA) to the virus like particles (VLPs) of some culturable HuNoVs, such as HuNoV genogroup I genotype 1 (GI.1), GII.3, GII.4 and GII.17 (Kilic et al. 2019). Murakami et al. demonstrated that GCDCA promoted GII.3 replication by enhancing endosomal uptake, endosomal acidification, activity of endosomal/lysosomal enzyme ASM, and ceramide levels on the apical membrane through S1PR2 in HIEs (Murakami et al. 2020). On the other hand, Kilic et al. found that BAs rendered/enhanced the binding of certain HuNoV genotypes (e.g., GII.1 and GII.10) with HGBA (Kilic et al. 2019). Lindesmith et al. also reported that bovine bile, but not GCDCA or TCA, enhanced the binding of VLPs of GII.2 Chapel Hill strain (CH) to pig gastric mucin III, which contained several secretor HBGAs, in a dose-dependent manner (Lindesmith et al. 2020). These results suggested that BAs had complex effects on host cells and/or HuNoV particles in a strain-dependent manner and influenced HuNoV life cycles. More systematic analyses of multiple BAs and genotypes of HuNoVs are warranted to better understanding effects of BAs on HuNoV replication.

Murine norovirus (MuNoV) grows in mouse macrophage and dendritic cell lines. Its infection of mice has been used as a model to study the replication and pathogenesis of noroviruses due to the lack of an animal model for HuNoVs with the exception of germfree piglet model (Cheetham et al. 2006; Karst and Tibbetts 2016; Karst et al. 2003; Wobus et al. 2006). CD300lf is a proteinaceous cellular receptor for MuNoV binding and entry into mouse cell lines, primary cells, and mice (Orchard et al. 2016).

The capsid protein of NoVs is divided to shell (S) and protruding (P) domains. P domains form dimers bind to the host receptors to initiate virus infection. There are 2 binding sites for GCDCA and LCA at P domain dimer interface of MuNoV. These BA binding sites are distinct from those for CD300lf receptor. GCDCA enhances intrinsic affinity of P domain of viral capsid protein for cellular receptor CD300lf and is necessary for cell attachment (Nelson et al. 2018). Subsequent studies showed that GCDCA and TCA caused the rotation and contraction of MuNoV P domain onto the viral capsid shell surface. This stabilized P domain appeared to allow for a higher degree of receptor saturation with virus (Sherman et al. 2019). In contradiction to the above results, CDCA and DCA may directly prime type III IFN induction in proximal gut, resulting in the inhibition of MuNoV replication in intestinal immune cells (Grau et al. 2020). These studies provided a biophysical characterization of MuNoV capsid-receptor and capsid-BA interactions and had important implications for the design of norovirus therapeutics.

Like HuNoVs, HuSaVs also cause acute gastroenteritis with similar transmission route and symptoms to HuNoV-associated illnesses (Oka et al. 2015). Recently, Takagi et al. reported the first efficient growth of multiple HuSaVs (GI.1, GI.2 and GII.3) in one human duodenum cell line HuTu80 in culture medium supplemented with BAs (GCA or GCDCA) (Takagi et al. 2020). This inexpensive and reproducible *in vitro* cell culture system can be further optimized to provide a fundamental scientific tool for HuSaV research and future infection control strategy development. These results suggest that BAs are essential for the successful propagation of certain human noroviruses and sapoviruses in cell culture. BAs may function *via* cells and/or the viral particles. However, the molecular mechanisms need to be investigated further.

## Roles of BAs in the replication of enteric rotavirus

Rotavirus is named for its classic “wheel-shaped” appearance by electron microscopy. Its positive sense RNA genome is double-stranded, consisting of 11 fragments. Rotavirus is the leading cause of severe gastroenteritis in children less than 5 years of age (Crawford et al. 2017) and also a common cause of diarrhea in young animals (Doro et al. 2015). Although effective live-attenuated vaccines are available for human rotavirus infection (Carvalho and Gill 2019), rotavirus still remains the most important cause of gastroenteritis in infants and children worldwide. Kim and Chang demonstrated that CDCA and DCA significantly reduced rotavirus replication in cell culture in a dose-dependent manner with activation of FXR/small heterodimer partner signaling pathway. Furthermore, a significant reduction in fecal rotavirus shedding was also detected between 1 and 3 d post inoculation in CDCA-fed mice (Kim and Chang 2011). This data may open a new avenue for the development of antiviral and/or disinfection for rotaviruses.

## Roles of BAs in the replication of enteric coronaviruses

Coronaviruses (CoVs) are positive sense, single-stranded RNA viruses that cause diseases in mammals and birds. They can cause respiratory, enteric, hepatic and neurological diseases, and peritonitis with highly variable severity (De Groot et al. 2012). Particularly notable are the beta-CoVs that cause enteric, respiratory and neurologic infections in cattle and swine and fatal respiratory disease in humans. Most famous of them are bovine CoV, porcine hemagglutinating encephalomyelitis virus (PHEV), severe acute respiratory syndrome (SARS), Middle East respiratory syndrome (MERS) and coronavirus disease 2019 (COVID-19) (Mora-Diaz et al. 2019; Saif and Jung 2020). Porcine epidemic diarrhea virus (PEDV) and porcine deltacoronavirus (PDCoV) are major causative agents of lethal watery diarrhea in neonatal piglets in the past decade (Jung et al. 2016; Koonpaew et al. 2019; Ma et al. 2015; Stevenson et al. 2013; Sun et al. 2012; Wang et al. 2014). Kim et al. isolated and passaged a PEDV strain in Vero cells in the presence of GCDCA, trypsin or elastase to obtain 8aa, KD or AA PEDV strains. PEDV 8aa strain grew to higher titer (> 8 log_10_ TCID_50_/mL) than KD and AA (< 7 log_10_ TCID_50_/mL) strains *in vitro* after the 20th passage level. Interestingly, replication of 8aa was inhibited by trypsin. It replicated poorly and was fully attenuated in nursing piglets (Kim et al. 2017). Su et al. reported that bile, CDCA, GCDCA, UDCA and DCA increased the infectivity of PEDV strain icPC22A in Vero and porcine small intestinal epithelial cell line (IPEC-DQ, a subclone of IPEC-J2), but had no or negative effects on PEDV variant icPC22A-S1Δ197, which lacks 197-aa in spike protein N-terminal domain (Su et al. 2019). Recently, we found that CDCA and LCA had antiviral activity against PDCoV replication in LLC-PK1 and IPEC-J2 cells. However, BAs GCDCA, CA, TCA, DCA, GDCA and TDCA had no effects on PDCoV replication. Further, we found that CDCA and LCA inhibited PDCoV replication at post-entry stages by inducing the production of IFN-λ3 and IFN-stimulated gene 15 (*ISG15*) *via* GPCRs in IPEC-J2 cells (paper submitted to Veterinary Microbiology). These results suggested that some BAs could affect enteric coronavirus replication. The interaction among BAs, microbiota and intestinal enzymes (e.g. proteases), BA effects and molecular mechanisms on coronavirus replication *in vivo* remain unclear. Such findings may also have implications for the human epidemic/pandemic CoVs (SARS-CoV-1, MERS-CoV and SARS-CoV-2) because all have been reported to infect the gastrointestinal tract (Leung et al. 2003; Wong et al. 2020; Zhou et al. 2017).

## Perspectives and future research

In addition to the modulation of enteric virus replication, BAs can limit *in vitro* replication of herpes simplex virus (Herold et al. 1999), human immunodeficiency virus (Lloyd et al. 1988), influenza A virus (Luo et al. 2018), mouse cytomegalovirus (Schupp et al. 2016), simian virus 40 (Kim et al. 1999), chikungunya virus (Winkler et al. 2020), and hepatitis D virus (Veloso Alves Pereira et al. 2015). On the contrary, it promotes *in vitro* replication of hepatitis B virus (Kim et al. 2010; Reese et al. 2013) and hepatitis C virus (Chang and George 2007; Chhatwal et al. 2012; Patton et al. 2011; Scholtes et al. 2008). These studies demonstrated that local BAs in liver, intestines and systemic BAs in blood, and different types of BAs play a complex role in the life cycle of different viruses.

BAs activate multiple signaling pathways and transcription factors to promote or inhibit virus replication *in vivo* and *in vitro*. It would be interesting to determine if other viruses that preferentially replicate in liver or intestines, such as hepatitis A virus, hepatitis E virus and other enteric viruses, are also affected by BAs and have evolved mechanisms to survive or even benefit from BA signaling. To date, studies have reported that BAs activated several key innate signaling pathways to potentiate antiviral immunity (Fiorucci et al. 2018; Grau et al. 2020; Hu et al. 2019). Therefore, the potential of using BAs to enhance innate antiviral responses and engage host immune system to clear infection may be a useful strategy for treatment of some hepatotropic and enteric virus infections. However, Podevin et al. demonstrated that CDCA inhibited antiviral activities of IFN-α in hepatic cells (Podevin et al. 1999). Grau et al. reported that MuNoV infection in proximal gut was inhibited by CDCA and DCA through type III interferon induction, while high expression levels of FXR simultaneously enhanced MuNoV infection in distal gut (Grau et al. 2020). These findings partially explain the complex role of different BAs in regulating aspects of enteric virus infections and should stimulate interest in further investigation of the role of BAs in virus replication, induction of innate immunity and microbiota-virus-host interactions.

## Conclusions

In addition to facilitating the absorption of dietary fats, BAs act as signaling molecules through different cell receptors and signaling pathways to regulate lipid, glucose and energy metabolism. Research in the past two decades has contributed substantially to our understanding of the role of BAs in virus infections. In this review, we have discussed BA biosynthesis, transport and metabolism and the mechanistic links between BAs and enteric virus infections, with a focus on enteric caliciviruses, rotaviruses, and coronaviruses. We also summarized the roles of certain BAs on replication of enteric viruses in Table 1. BAs, BA-activated receptors and signaling pathways could be therapeutic targets for the development of antiviral drugs to treat enteric virus infections.

**Table 1:** Summary of bile acids on the replication of enteric viruses

Viruses	Major virus replication sites (D, J, I, or C) in vivo	In vitro cell line	Name of BAs (concentration tested)			References
			Pro-viral	Anti-viral	No effects	
PoSaV/GIII/Cowden strain (or PEC)	D, J	LLC-PK1	GCA (50–500 μM), GCDCA (50–500 μM), TCDCA (50–500 μM), GDCA (100 μM) , TDCA (50–500 μM) and TLCA (100–500 μM)	No	UDCA and TUDCA (10–500 μM)	Chang et al. 2004
			GCDCA (100 μM)	Not tested	Not tested	Shivanna et al. 2014, 2015
HuNoV/GII.3	D, J, I	Duodenum HIE (D104 line)	GCDCA (500 μM)	Not tested	Not tested	Murakami et al. 2020
		Jejunal HIE (J3 line)	GCDCA (500 μM)	Not tested	Not tested	
		Jejunal HIE (J2 line)	GCA (500 μM), GDCA (500 μM), GCDCA (5–500 μM), TCA (500 μM), TDCA (500 μM), TCDCA (5–500 μM), TLCA (500 μM), TUDCA (500 μM), CA (500 μM), CDCA (100 μM), DCA (100 μM) and LCA (50 μM)	No	UDCA (500 μM)	
		Ileum HIE (IL104 line)	GCDCA (500 μM)	Not tested	Not tested	
MuNoV/GV.1	J, I, C	BV2	GCDCA (500 μM)	No	TCA (500 μM), GCA (500 μM), CA (500 μM) and TDCA (500 μM)	Nelson et al. 2018
		CMT93	No	CDCA (100–200 μM) and DCA (100–200 μM)	CA (50–200 μM) and LCA (50–200 μM)	Grau et al. 2020
HuSaV/GI.1	D, J	HuTu80	CA (125 μM), GCA (1000 μM), GCDCA (500 μM)	No	DCA (71.4 μM)	Takagi et al. 2020
		NEC8	CA (250 μM), GCA (500 μM), GCDCA (200 μM)	No	DCA (20 μM)	
HuSaV/GI.2		HuTu80	GCA (1000 μM)	Not tested	No tested	
HuSaV/GII.3		HuTu80	CA (125 μM), GCA (1000 μM), GCDCA (500 μM)	No	DCA (71.4 μM)	
Rotavirus	J, I	MA104	No	CDCA (100 μM) and DCA (100 μM)	CA (200 μM), UDCA (200 μM) and GCDCA (200 μM)	Kim and Chang 2011
		Caco-2	Not tested	CDCA (200 μM)	Not tested	
PEDV	J, I	Vero	GCDCA (100 μM)	Not tested	Not tested	Kim et al. 2017
		Vero	CDCA (25–200 μM), GCDCA (50–200 μM), UDCA (50–200 μM) and DCA (50–200 μM)	No	No	Su et al. 2019
		IPEC-DQ	CDCA (100–200 μM), GCDCA (25–200 μM), UDCA (50–200 μM) and DCA (50–200 μM)	No	No	
PDCoV	J, I	LLC-PK1	No	CDCA (100 μM), LCA (12.5 μM)	GCDCA (100 μM), CA (100 μM), TCA (100 μM), DCA (50 μM), GDCA (100 μM), and TDCA (100 μM)	unpublished data
		IPEC-J2	No	CDCA (100 μM), LCA (6 μM)		

Notes: “No” means BAs tested in the study have no indicated effects; “Not tested” means no other BAs were tested in the study. *CA* cholic acid, *DCA* deoxycholic acid, *LCA* lithocholic acid, *CDCA* chenodeoxycholic acid, *TCA* taurocholic acid, *TCDCA* taurochenodeoxycholic acid, *GCA* glycocholic acid, *GCDCA* glycochenodeoxycholic acid, *UDCA* ursodeoxycholic acid
